# Biofilm Spreading by the Adhesin-Dependent Gliding Motility of *Flavobacterium johnsoniae*: 2. Role of Filamentous Extracellular Network and Cell-to-Cell Connections at the Biofilm Surface

**DOI:** 10.3390/ijms22136911

**Published:** 2021-06-27

**Authors:** Keiko Sato, Masami Naya, Yuri Hatano, Naoki Kasahata, Yoshio Kondo, Mari Sato, Katsuki Takebe, Mariko Naito, Chikara Sato

**Affiliations:** 1 Department of Microbiology and Oral Infection, Graduate School of Biomedical Sciences, Nagasaki University, Nagasaki 852-8588, Japan; mnaito@nagasaki-u.ac.jp; 2 Health and Medical Research Institute, National Institute of Advanced Industrial Science and Technology (AIST), Tsukuba 305-8566, Japan; masami.naya@gmail.com (M.N.); iyuhri@gmail.com (Y.H.); naoki.kasahata@gmail.com (N.K.); ma-satou@aist.go.jp (M.S.); 3 Department of Pediatric Dentistry, Graduate School of Biomedical Sciences, Nagasaki University, Nagasaki 852-8588, Japan; yosioji@nagasaki-u.ac.jp; 4 Oral and Maxillofacial Surgery II, Graduate School of Dentistry, Osaka University, Yamadaoka, Suita, Osaka 565-0871, Japan; u389521f@ecs.osaka-u.ac.jp

**Keywords:** focal adhesion complex, extracellular fibers, vesicle, cell-to-cell connections, transmission electron microscopy, Grid Stamp-Peel method, antibiotics resistance

## Abstract

*Flavobacterium johnsoniae* forms a thin spreading colony on nutrient-poor agar using gliding motility. As reported in the first paper, WT cells in the colony were sparsely embedded in self-produced extracellular polymeric matrix (EPM), while *sprB* cells were densely packed in immature biofilm with less matrix. The colony surface is critical for antibiotic resistance and cell survival. We have now developed the Grid Stamp-Peel method whereby the colony surface is attached to a TEM grid for negative-staining microscopy. The images showed that the top of the spreading convex WT colonies was covered by EPM with few interspersed cells. Cells exposed near the colony edge made head-to-tail and/or side-to-side contact and sometimes connected via thin filaments. Nonspreading *sprB* and *gldG* and *gldK* colonies had a more uniform upper surface covered by different EPMs including vesicles and filaments. The EPM of *sprB*, *gldG*, and WT colonies contained filaments ~2 nm and ~5 nm in diameter; *gldK colonies* did not include the latter. Every cell near the edge of WT colonies had one or two dark spots, while cells inside WT colonies and cells in SprB-, GldG-, or GldK-deficient colonies did not. Together, our results suggest that the colony surface structure depends on the capability to expand biofilm.

## 1. Introduction

*Flavobacterium johnsoniae* is an aerobic Gram-negative rod-shaped bacterium that uses gliding motility to move rapidly over solid surfaces and thereby forms thin spreading colonies on agar [1,2]. The cell surface adhesin SprB forms filaments and is a component of the motility machinery of *F. johnsoniae* [3]. The movement of SprB along a closed helical loop track on the cell surface causes the gliding motility of a cell [4,5,6]. RemA is also part of the gliding machinery because it is known to allow some gliding in the absence of SprB [7]. Colony spreading of *F. johnsoniae* is influenced by modifications of the motility machinery complex, such as deficiency of SprB or RemA, and by environmental factors, such as the moisture content of the medium and the type of nutrient available. For example wild-type (WT) *F. johnsoniae* forms thin film-like spreading colonies on nutrient-poor agar medium, while *sprB* deletion mutant (*sprB*) cells form small nonspreading colonies [1]. The upper surface of WT colonies is convex, whereas the upper surface of *sprB* colonies is flatter [1].

SprB and other cell surface components of the gliding motility machinery are translocated to the cell surface or secreted by the Bacteroidetes-specific type IX secretion system (T9SS) [8]. The T9SS includes the core components GldK/PorK, GldL/PorL, GldM/PorM, and GldN/PorN, which are interconnected with the gliding motility machinery [8,9,10]. Therefore, T9SS-deficient mutants, including the *gldK* mutant, form nonspreading colonies [11]. Other secreted T9SS cargo proteins include many extracellular or cell surface enzymes, adhesins, and virulence factors [12,13].

In addition to the cell surface adhesin, T9SS (GldK, GldL, GldM, GldN, SprA, SprE, and SprT) and gliding motility proteins (GldA, GldB, GldD, GldF, GldG, GldH, and GldI) are involved in the formation of spreading colonies. The non-gliding bacterium *Porphyromonas gingivalis* harbors the T9SS proteins but not these orthologous gliding motility proteins. GldF and GldG are membrane proteins that appear to interact with GldA to form an ABC transporter that is required for gliding. In *F. johnsoniae*, GldG protein was considered to be necessary for gliding motility [14]. In *Flavobacterium psychrophilum,* disruption of the *gldG* gene results in a dramatic reduction of GldJ abundance and provokes a remarkable diminution of GldK, GldN, and SprT proteins [15].

Biofilms are known to be communities of surface-attached microorganisms embedded in a self-produced extracellular polymeric matrix (EPM) [16,17]. In *Staphylococcus aureus* and *Cutibacterium acnes* (previously known as *Propionibacterium acnes*) biofilms, the cells are embedded in self-produced EPM containing eDNA and proteins as well as exopolysaccharide [18,19]. In the *Flavobacterium* genus, the fish pathogen *Flavobacterium psychrophilum* forms a biofilm when it colonizes the gill [20,21,22]. Scanning confocal laser microscopic observations show that most live bacterial cells are found in the deeper and intermediate layers, while dead cells predominate in the remaining biofilm zone [21].

Biofilm formation and T9SS activity are related to the pathogenesis of many infectious diseases [14,23,24,25]. Indeed, biofilms are involved in many chronic human diseases, including periodontal diseases, some lung diseases, and infectious diseases [26,27].

The T9SS is used to secrete many potent virulence factors of human infectious diseases of the phylum *Bacteroidetes*, which includes periodontal pathogens, such as *Porphyromonas gingivalis* [8], *Tannerella forsythia* [Narita Y et al. 2014], and *Prevotella melaninogenica* [Kondo Y et al. 2018]. *P. gingivalis* shares some features with fish pathogens [24,25,28] and *F. johnsoniae* [8,9]. This makes *F. johnsoniae* a good model system for studying the behavior of pathogenic *Bacteroidetes* bacteria or the evolution of their pathogenicity. Cells of the gliding bacterium *Capnocytophaga gingivalis* present in the human oral microbiome carry polymicrobial cargoes, including nonmotile bacteria species, to new locations [29], suggesting that the gliding motility of a bacterium can contribute to the expansion of other species, including biofilm. Some members of the *Bacteroidetes* phylum that exhibit gliding motility, e.g., *Capnocytophaga canimorsus, Flavobacterium columnare*, and *Flavobacterium psychrophilum*, cause infectious diseases in humans and fish [30,31,32,33]. These reports suggest that the gliding motility of the biofilm-forming bacteria might influence their virulence via expansion of the biofilm.

Biofilm formation thus contributes to the virulence of bacteria and also influences their resistance to antibiotics [34,35,36,37]. To understand the mechanisms by which biofilm forms and expands, it is necessary to know the physical structure of biofilm in detail. However, the required structural analyses are lacking, especially for the biofilm formed at the interface between air and wet solid surfaces by bacteria such as *F. johnsoniae* (WT).

In the first part of this study, we investigated the internal structure of *F. johnsoniae* colonies spreading on nutrient-poor agar media using Epon-embedded thin-sectioning and transmission electron microscopy (TEM) [38]. In the spreading WT colonies, the cells were embedded in a self-secreted matrix that contained a thick filamentous network and vesicles, indicating biofilm formation [38,39]. The cell density close to the bottom of the colony was higher than in middle regions of the colony. By contrast, in nonspreading *sprB* colonies, the cells were tightly packed and surrounded by fewer intercellular substances, including vesicles, indicating immature biofilm [38]. However, what is the structure of the biofilm surface?

In this study, we characterized biofilm expansions involving gliding motility and revealed the surface structures of *F. johnsoniae* using a newly developed Grid Stamp-Peel method. To further investigate whether the biofilm forming ability of a colony is related to its antibiotic resistance, we performed antimicrobial susceptibility testing for various mutations.

## 2. Results

### 2.1. Grid Stamp-Peel Method

**Figure 1:**
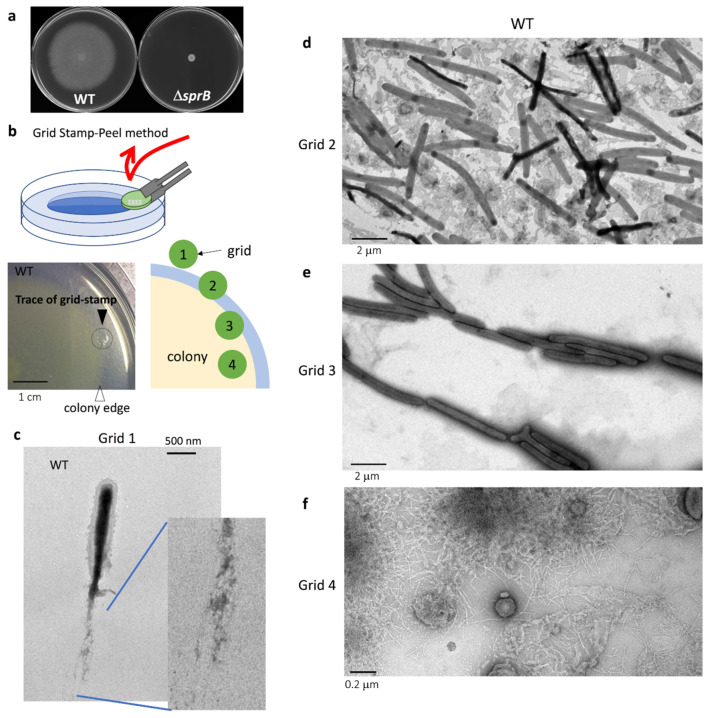
Grid Stamp-Peel peel method and the resulting TEM images of the top surface above the leading tip of a WT *F. johnsoniae* colony. The colony was formed on 1% A-PY2. (**a**) Colony spreading of *F. johnsoniae* on 1% agar-PY2 (peptone-yeast extract) medium (1%A-PY2) in a 9 cm diameter dish (5 days). Left panel, WT; right panel, *sprB*. (**b**) The Grid Stamp-Peel method (grid stamp method). Top panel: Schematic showing a colony being ‘stamped’ by a thin-carbon TEM grid. The surface layer of the colony is transferred to the carbon grid by pressing (stamping) the grid onto it. In practice, the weight of the grid provides sufficient pressure. Bottom panels: Expanding colony sampled by the grid stamp method (left) and a schematic indicating the stamping procedure (right). The *F. johnsoniae* colony has a yellow body and a surrounding translucent fringe as the leading edge. These are colored yellow and blue, respectively, in the diagram. Green circles indicate the position of each grid stamp (Grids 1 to 4). (**c**–**f**) TEM of grid stamps 1–4 stained with uranyl acetate. The structure of the colony surface varied, depending on the region sampled. (**c**) A single *F. johnsoniae* cell outside the extending translucent colony tip on the agar surface (position of Grid 1). Inset: enlargement (3×) of the indicated area. The leading cells laid a path of filaments. (**d**) Cells at a leading translucent fringe of the colony (position of Grid 2). Cells were interspersed among many small vesicles. (**e**) Cells immediately inside the edge of the colony (position of Grid 3). Cells made head-to-tail and/or side-to-side contact. (**f**) Images of the more proximal surface, 2 mm inside the yellow edge of the colony body (position of Grid 4). The surface was occupied by a substance containing extracellular fibers and vesicles. No cells were found.

To examine the wet surface of a colony on the agar plate, a glow-discharged carbon film on a TEM mesh grid was lightly pressed (stamped) onto the colony surface and was removed together with some surface cells and matrix structures (Figure 1b). The specimen grid was stained with uranyl acetate and imaged by TEM. This new method, which we call the ‘Grid Stamp-Peel method’, is a type of negative-stain TEM that allows the surface structures of biofilm formed on a wet substrate to be imaged.

### 2.2. The Surface Structure at the Leading Edge of Spreading WT Colonies

Using the Grid Stamp-Peel method, a glow-discharged thin carbon grid (see Materials and Methods 4.2 for details) was first stamped outside the leading edge of an expanding WT colony grown on nutrient-poor 1% agar PY2 (peptone-yeast extract) medium (1% A-PY2) (Figure 1b bottom right, Grid position 1). In this region, single *F. johnsoniae* cells laid a path of filaments (Figure 1c), and the area surrounding the cell was sometimes covered by a poorly-stained filamentous matrix. Images of the ‘grid stamps’ at the leading edge of the WT colony (Grid position 2–3) revealed that the space between cells was occupied by a matrix containing extracellular fibers and small (~30 nm) vesicles (Figure 1d–e). In the translucent area outside the yellow colony body (Figure 1b Grid position 2,d), the cells were attached to budding vesicles and also surrounded by many secreted vesicles and filaments. The cells were sometimes dispersed within the matrix (Figure 1d) or made head-to-tail and/or side-to-side contact. Near the edge of the colony body, more cells made such contacts, forming thick lines (Figure 1b Grid position 3,e). At the internal surface near the center of the colony, almost no bacterial cells were found (Figure 1b Grid position 4,f). The surface structures observed at each grid position are described more precisely in the following sections.

### 2.3. Comparison of the Surface Structure at the Edge of WT and Adhesin sprB-Deficient Colonies

**Figure 2:**
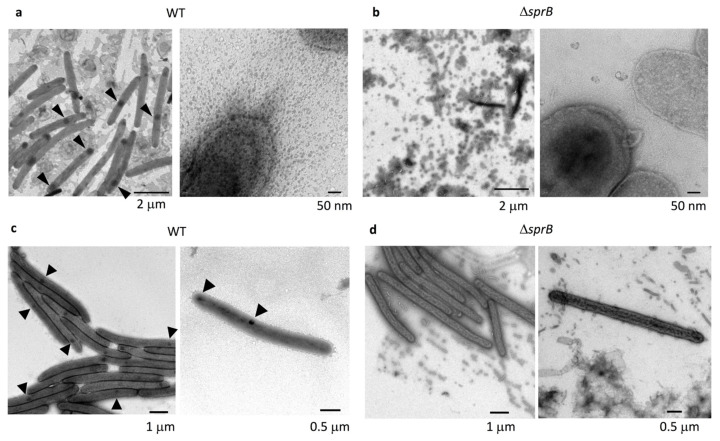
Surface structures at the edge of a WT and a *sprB* colony. Colony surface at the translucent leading edge of a WT colony was sampled using the grid stamp method (position of Figure 1b, Grid 2), negatively stained, and imaged by TEM. (**a**,**c**) WT and (**b**,**d**) *sprB* colonies on 1% A-PY2. (Left) Low magnification images. (Right) Higher magnification images. (**a**) WT cells were interspersed or (**c**) made head-to-tail and/or side-to-side contact. In both cases, the cells were surrounded by many small vesicles and filaments. (**b**,**d**) *sprB* cells at the edge of the small nonspreading colony were found interspersed among thin extracellular fibers and a small number of vesicles. One or two dark spots (arrowheads) were seen in each WT cell (**a**,**c**), but not in *sprB* cells (**b**,**d**).

**Figure 3:**
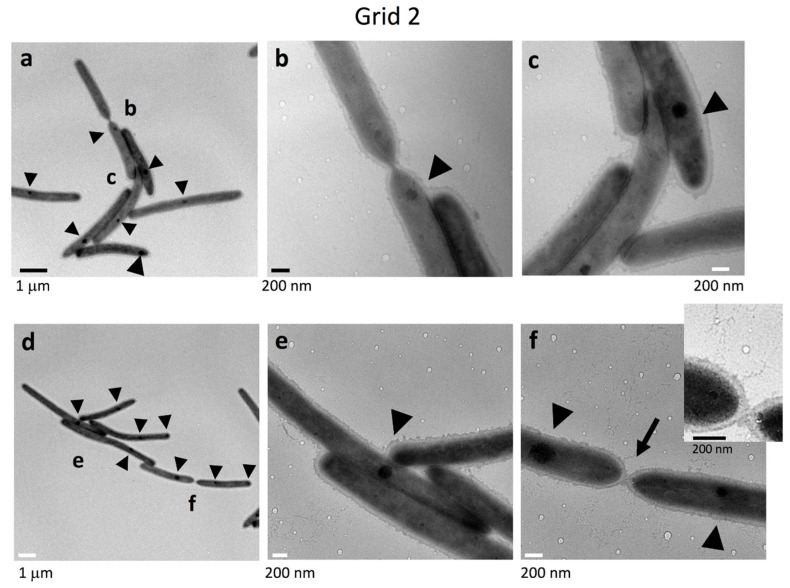
Cell connections at the translucent edge of a typical spreading WT colony. The colony surface was sampled using the grid stamp method at Grid position 2 (Figure 1b), and connections between cells in clusters were imaged by negative-stain TEM. (**a**) Low magnification image. (**b**,**c**) Higher magnification images of the annotated areas in (**a**). (**d**–**f**) Another cell cluster. (**d**) Low magnification image. (**e**,**f**) Higher magnification images of the annotated areas in (**d**). (**f**) Many cells made head-to-tail and/or side-to-side contacts. Inset: enlargement (1.7×) of the connection indicated by an arrow. The area surrounding cells was covered by poorly stained filamentous matrices and vesicles as shown in (**f**). Dark spots of high electron density in the cells are indicated by arrowheads. One or two dark spots of high electron density are distinguishable for each cell.

The translucent leading edges (Grid position 2) of the WT colonies were intensively imaged using the grid stamp method and negative-stain TEM. The cells were dispersed among the matrix (Figure 2a, left) or clustered making head-to-tail and/or side-to-side contact (Figure 2c, left). At higher magnification, the space between cells was covered by many vesicles and thin extracellular fibers (Figure 2a,c, right). Next, we imaged nonspreading yellow colonies formed by *sprB.* These had a well-defined edge and lacked the translucent region observed for spreading colonies (compare Figure 1a, left, Figure 1b, bottom left, and Figure 1a, right). At the edge of *sprB* colonies, the cells gathered but did not form tight connections (Figure 2d, left). The space between the cells was covered by a small number of vesicles and thin extracellular fibers (Figure 2b,d, left). The vesicle density (number of vesicles per unit area; Figure 2b,d, right) was clearly smaller than for the WT colonies (Figure 2a,c, right). Furthermore, every WT cell had one or two dark spots of high electron density stained by uranyl acetate (Figure 1c, Figure 2a,c and Figure 3a, arrowheads), but *sprB* cells did not (Figure 2d).

### 2.4. Cell Connections at the Translucent Edges of Spreading WT Colonies

The connections between the cells in clusters at Grid position 2 (Figure 1b) were precisely imaged at higher magnification (Figure 3). Cells with internal dark spots made head-to-tail and/or side-to-side contacts forming clusters. They were surrounded by a poorly stained filamentous matrices and vesicles. Head-to-tail contact occurred via a single connection point at the interface, and the two cells were also connected by very thin filaments emanating from their surface (Figure 3b,f), suggesting that the filaments might be involved in cell connectivity. This idea would at least partially explain why single cells tend to follow cell clusters and the specific paths followed by cell clusters observed using time-lapse fluorescence microscopy (First part of this paper: Figure 1c upper and Figure S2a of [38]).

### 2.5. Surface Structure Inside the Edge of WT Colony Bodies

**Figure 4:**
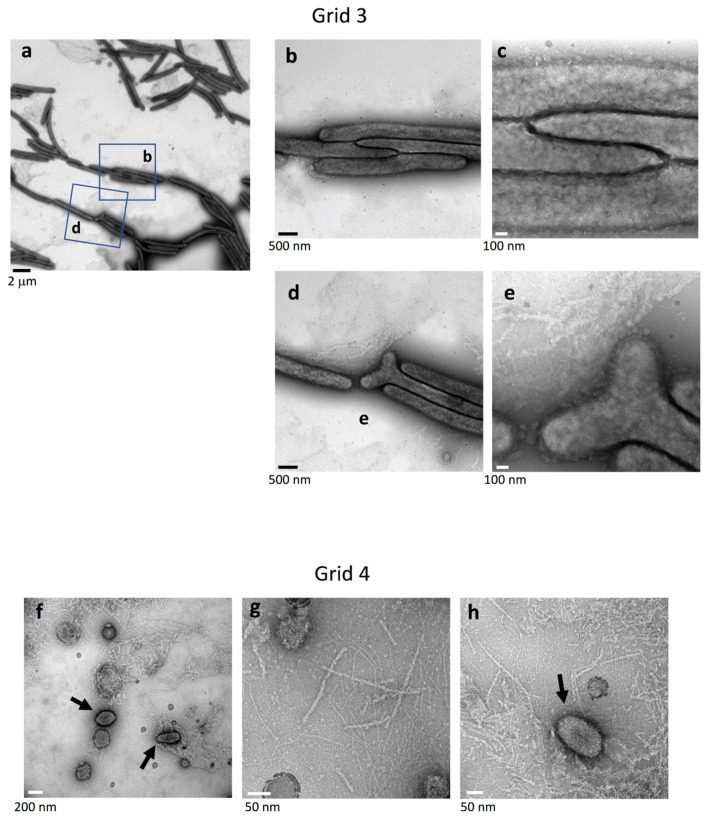
Top surfaces inside the edges of a WT colony body. Colony surfaces were sampled using the grid stamp method at Grid positions 3 and 4 (Figure 1b) and imaged by negative-stain TEM. (**a**) Low magnification image of the top surface slightly inside the edge of the yellow colony body (Grid position 3). Head-to-tail and/or side-to-side close contacts were formed between a large number of cells. (**b**) Higher magnification image (3.7×) of the area indicated in (**a**). (**c**) Higher magnification image (3×) of the central area in (**b**). (**d**) Higher magnification image (3.7×) of the area indicated in (**a**). (**e**) Higher magnification image (3×) of the central area in (**d**). (**f**–**h**) Top surfaces 2 mm inside the edge of the yellow colony body (Grid position 4). (**f**) Low magnification image. Almost no cells were imaged, but several large vesicles are prominent. (**g,h**) Higher magnification images. Most areas are occupied by large and small vesicles and extracellular filaments of various diameters. The large vesicles (150–250 nm in diameter) are indicated by arrows.

In the more proximal surface slightly inside the yellow body of WT colonies (Grid position 3), more cells made close head-to-tail and/or side-to-side contact (Figure 4a). Dark spots of high electron density were rarely observed or only faintly visible in the cells (Figure 1e and Figure 4a–c). The bacterial cells were surrounded by a poorly stained filamentous matrix and vesicles (Figure 4b,c). Occasionally, bacterial cells with a bifurcated end (pole) were observed (Figure 4d,e). In contrast, cells were rare 2 mm inside the edge of the colony body (Grid position 4), but the surface was occupied by extracellular fibers of various diameter (2–8 nm diameter) and large, medium, and small vesicles (Figure 1f and Figure 4f–h). The large vesicles (150–250 nm in diameter), medium-sized vesicles (~30 nm), and many visible small vesicles were circular or elliptical (arrows), and the remaining area was occupied by a matrix containing filaments and particles (Figure 4d–f). The larger (5–8 nm in diameter) filaments were dispersed in a network of smaller filaments (<2 nm in diameter), presumably reflecting the presence of a rich EPM covering the top of the WT colony.

### 2.6. Top Surface Inside the Edge of Nonspreading sprB Colonies

**Figure 5:**
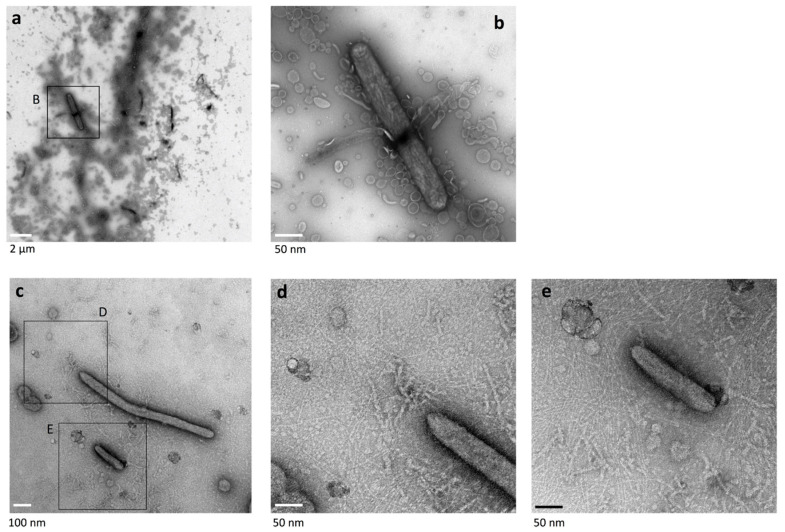
Colony surface inside the edge of a typical *sprB* colony imaged using the grid stamp method. (**a**) Low magnification image. (**b**) High magnification image (4.7×) of the annotated square in (**a**). (**c**) Medium magnification image of another area. (**d**,**e**) High magnification images (3×) of the annotated squares in (**c**). Just a small number of cells were observed, but most areas were occupied by thin extracellular fibers and vesicles. The vesicles had various shapes and sizes: small vesicles <30 nm, medium-sized vesicles (~ 30 nm), and a limited number of larger circular vesicles (150–200 nm in diameter).

Grid stamp was carried out inside the edge of *sprB* colonies and imaged by negative-stain TEM. Like for the WT, thin and thick extracellular fibers and small vesicles were present (Figure 5). A small number of cells were dispersed among them; the cells did not have dark spots of high electron density. A limited number of large round vesicles (150–250 nm in diameter) and medium-sized round vesicles (~30 nm) were also observed. In contrast to the spatial variations observed on the surface of WT colonies, the surfaces inside and at the edge of the nonspreading *sprB* colonies were similar.

### 2.7. Surface Structure of Nonspreading gldK Mutant Colonies Deficient in T9SS

**Figure 6:**
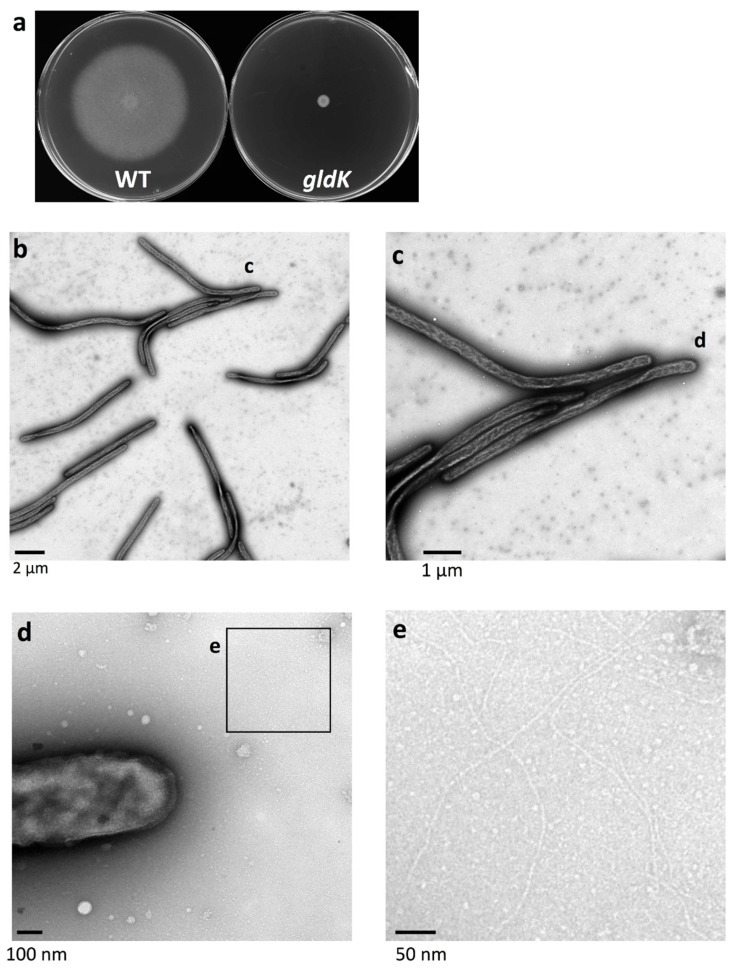
Colony surface at the edge of nonspreading *gldK* mutant colonies imaged using the grid stamp method. (**a**) Colony spreading of *F. johnsoniae* on 1% A-PY2 in a 9 cm diameter dish (cultured for 5 days). Left panel, WT; right panel, *gldK* mutant. (**b**) Low magnification grid stamp negative-stain TEM image recorded at the edge of a typical nonspreading *gldK* mutant colony. (**c**) Higher magnification image (2.5×) of the annotated area in the preceding panel. (**d**) Higher magnification image (5.4×) of the area indicated in (**c**). (**e**) Higher magnification image (3.3×) of the central area in (**d**). The cells made head-to-tail and/or side-to-side contact, like cells in the WT colony. The space between the cells was occupied by thin fibers and small vesicles but was without thick fibers (5–8 nm in diameter) and large vesicles (150–250 nm).

T9SS cargo proteins, including SprB, are not translocated to the cell surface in T9SS-deficient *F. johnsoniae* mutants [8,11,13]; *gldK* mutant cells deficient in T9SS form a nonspreading colony [11]. The surface at the edge of *gldK* mutant colonies was observed using grid stamp and negative-stain TEM. The cells made head-to-tail and/or side-to-side contact (Figure 6), which is similar to the cells of WT colonies but different from cells of *sprB* colonies. Thin fibers and vesicles were dispersed around the *gldK* mutant cells. However, neither large circular vesicular structures (150–250 mm) nor thick extracellular fibers (5–8 nm in diameter) were found, in contrast to the WT and the *sprB* (Figure 1, Figure 2, Figure 3, Figure 4 and Figure 5). Furthermore, like for *sprB* colonies (Figure 2d), the dark spots imaged in WT cells (Figure 1d, Figure 2a,c and Figure 3, arrowheads) were not observed in the cells at the edge of *gldK* mutant colonies (Figure 6b–d).

### 2.8. Surface Structure of Nonspreading gldG Mutant Colonies Deficient in Gliding Motility Protein

**Figure 7:**
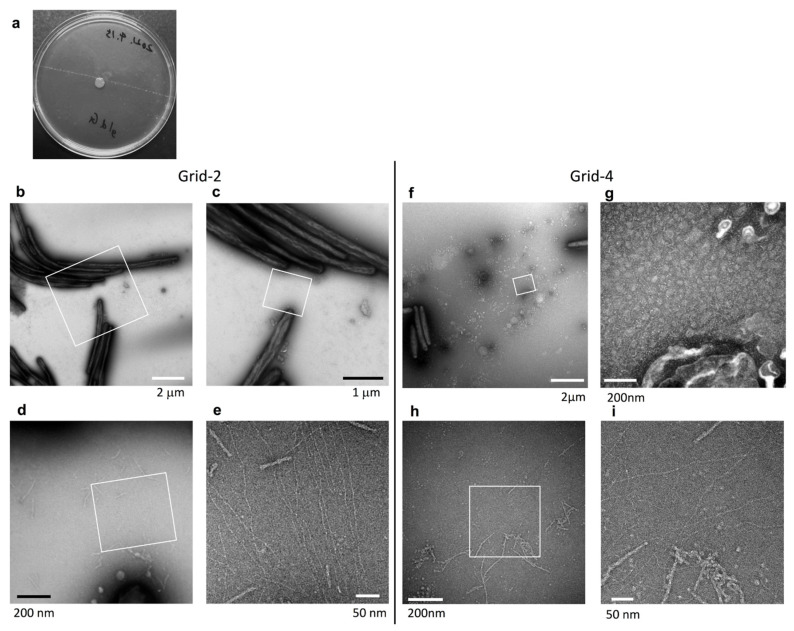
Colony surface of nonspreading *gldG* mutant colonies imaged using the grid stamp method. (**a**) Colony spreading of *gldG* mutant on 1% A-PY2 as in Figure 6. (**b**–**e**) Colony surfaces were sampled using the grid stamp method at the edge of the colony body (Grid position 2). (**b**) Low magnification grid stamp image recorded at the edge of a typical nonspreading *gldG* mutant colony. (**c**) Higher magnification image of the white square in the preceding panel. (**d**) Higher magnification image of the square indicated in (**c**). (**e**) Higher magnification image of the square in (**d**). Although the cells made head-to-tail and/or side-to-side contact like cells in the WT and *gldK* colonies. The space between the cells was occupied not only by thin fibers and small vesicles but also by thick fibers (5–8 nm in diameter) and large vesicles (150–250 nm), which are missing in the images of the *gldK* colony. (**f**–**i**) Top surfaces 2 mm inside the edge of the nonspreading *gldG* mutant colony (Figure 1b, Grid position 4). (**f**) Low magnification image. (**g**) Higher magnification image of the square in (**f**). (**h**) Low magnification image of another area. (**i**) Higher magnification image of the square in (**h**). Almost no cells were imaged, but small vesicles are prominent. (**g**–**i**) Higher magnification images. The area was occupied by small vesicles and fibers.

The products of *gldA*, *gldF*, and *gldG* form a complex that functions as an ATP-dependent transporter that is required for gliding [14]. The *gldG* mutant cells, deficient in gliding motility protein, form a nonspreading colony [14]. The *gldG* mutant colonies were examined using grid stamp and negative-stain TEM. At the surface of the colony edge (Figure 1b, Grid position 2), cells made head-to-tail and/or side-to-side contact (Figure 7b,c), which is similar to the cells of WT and *gldK* colonies. The space between the cells was occupied not only by thin fibers and small vesicles but also by thick fibers (5–8 nm in diameter) and large vesicles (150–250 nm), which were missing in the images of the *gldK* colony (Figure 6d,e). At the top surfaces 2 mm inside the edge of the colony (Figure 1b Grid position 4), almost no cells were imaged among vesicles, which is similar to the colonies formed by WT and *sprB* cells, but a smaller number of thick fibers are prominent (Figure 7h,i). Thick fibers are found on the colony of WT, *sprB*, and *gldG*, but not *gldK*. These results suggest that matrix production and cell localization on the colony surfaces were influenced by both cell surface adhesin SprB-dependent gliding and the T9SS as well as the gliding motility proteins.

### 2.9. Quantification of Biofilms Formed

**Figure 8:**
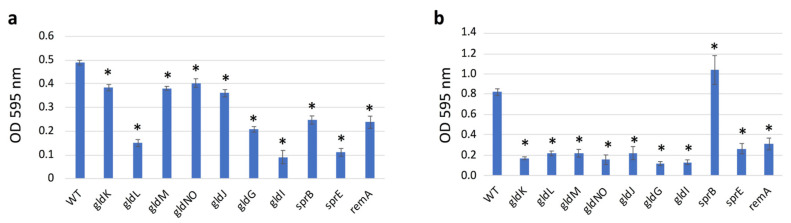
Crystal violet biofilm assay of WT, T9SS mutants (*gldK*, *gldL*, *gldM*, *gldNO*, and *sprE*), gliding motility proteins mutants (*gldG*, *gldI*, and *gldJ*), and adhesin proteins mutants (*sprB* and *remA*) strains. Cells were cultured in (**a**) PY2 broth or (**b**) CYE broth in a 24-well polystyrene plate for 24 h. The cells formed biofilm on the walls of the wells. Bar chart represents the results of the crystal violet microtiter biofilm assay. Vertical axis: optical density at 595 nm with the standard deviation. All the tested mutants produced biofilm in both nutrient-poor PY2 and nutrient-rich CYE medium. The amounts of biofilm produced by WT and *sprB* mutants are larger than those by the other mutants in CYE. The plate assay was performed three times for all strains; the averages and standard deviations are indicated. Asterisks denote Student’s *t*-test significance compared with WT (* *p* < 0.05).

When cultured on glass slides immersed in liquid medium, the fish pathogen *Flavobacterium columnare* [40] forms mature biofilms that include extracellular polymeric substances (EPS) and water channels [21]. To find out whether *F. johnsoniae* also forms biofilms on a substrate immersed in liquid medium, the WT and its mutant cells were individually inoculated onto static nutrient-rich CYE or nutrient-poor PY2 liquid media in 24-well polystyrene assay plates (Figure 8). The cells grew and formed biofilm on the surface of the wells. These biofilms were then measured using the crystal violet microtiter biofilm assay to obtain a quantitative evaluation. Thus, the biofilm forming abilities of all were confirmed, indicating that *F. johnsoniae* forms a biofilm on PY2 medium regardless of its T9SS or gliding ability, as suggested in the first part of this study [38]. In PY2, the amount of biofilm formed by WT was larger than the amounts formed by T9SS mutants (*gldK*, *gldM*, and *gldNO*), gliding motility proteins mutants (*gldG* and *gldJ*), and adhesin proteins mutants (*sprB* and *remA*) strains, respectively (Figure 8a). However, the biofilm amount formed by WT was clearly larger than the amount formed by *gldL, gldI*, and *sprE*, respectively. In CYE, the amount of biofilm produced by WT and *sprB* mutants was clearly larger than the amount formed by the other mutants, respectively (Figure 8b). It might suggest the specialty of *sprB* among the mutants.

### 2.10. Antibiotic Susceptibility

**Figure 9:**
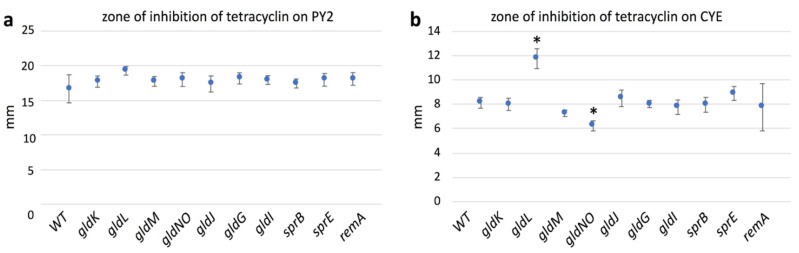
Antimicrobial effect in disc diffusion assay of WT and the mutants. Measurements of diameters of the inhibition zones of WT and the mutants against tetracycline. The plates of the disk diffusion test were incubated in (**a**) PY2 broth or (**b**) CYE medium for 2 days at 25 °C. The averages and standard deviations were indicated (*n* = 4). Asterisks denote Student’s *t*-test significance compared with WT (* *p* < 0.05).

**Figure 10:**
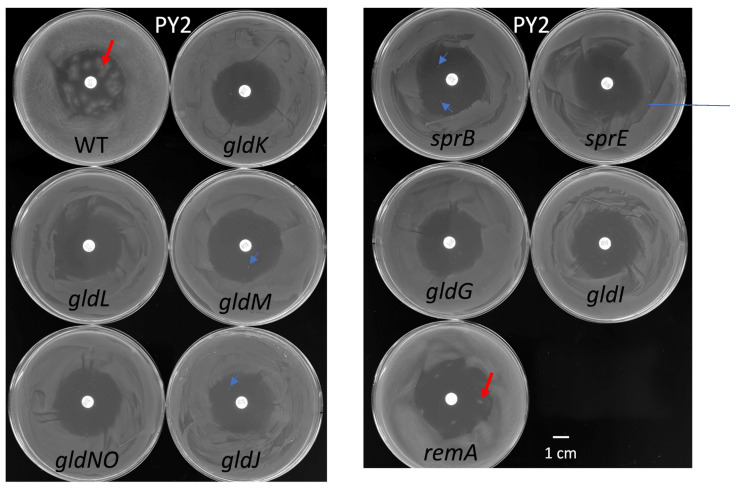
The antibiotic resistance of WT and the mutants in diffusion test. For the disk diffusion test, the PY2 plates were incubated at 25 °C for 5 days. Spreading colonies of *F. johnsoniae* WT and *remA* mutant were clearly found as isolated colonies within the inhibition zone (red arrows), although the colonies of *remA* were smaller than those of WT. One or two very small rigid colonies were found within the inhibition zone of *gldM*, *gldJ*, and *sprB* mutants (blue arrows).

Tetracycline and its derivates are the primary drugs used in periodontal treatment. To assess the resistance against antibiotics, *F. johnsoniae* WT, T9SS mutants (*gldK*, *gldL*, *gldM*, *gldNO*, and *sprE*), gliding motility proteins mutants (*gldG*, *gldI*, and *gldJ*), and adhesin proteins mutants (*sprB* and *remA*) strains were subjected to the disc diffusion method on PY2 medium (Figure 9a). The average diameters of the inhibition zones representing their susceptibilities were all comparable in size. After five days of culture, isolated spreading colonies of *F. johnsoniae* WT and *remA* mutant were clearly imaged within the inhibition zone (Figure 10, red arrow). Because colonies deficient in mobile cell surface adhesin RemA [7] formed several small spreading colonies, the antibiotic resistance of *remA* is suggested, which might reflect differences between adhesin *remA* and *sprB.* One or two very small rigid colonies were found within the inhibition zone of *gldM, gldJ*, and *sprB* mutants (blue arrow). The strains forming colonies within the inhibition zone produced relatively large amount of biofilm when they were cultured on glass slides immersed in PY2 medium (Figure 8). All the strains were next subjected to the disc diffusion method on CYE medium. The diameter of inhibition zone in *gldL* mutant were larger than those of the other mutant strains and WT (Figure 9). After five days of culture, no colony was found for any of the strains of *F. johnsoniae* within the inhibition zone on the CYE medium in the plates, which is in contrast to the results on PY2 medium.

## 3. Discussion

**Figure 11:**
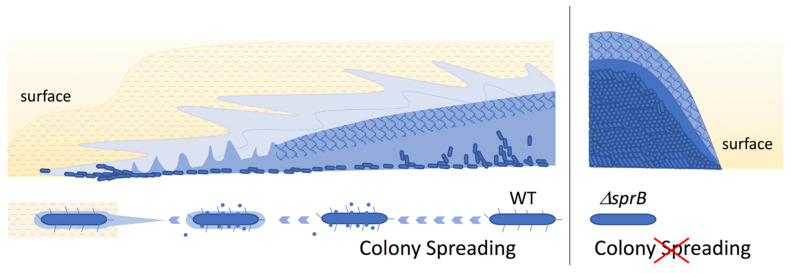
Schema of bacterial biofilm expansion by gliding motility. (Left panel) Outside the spreading lucent edge of WT colonies, single leading cells lay a path of filaments and vesicles. At the edge of the translucent fringe, many of the cells present make head-to-tail and/or side-to-side contact, and there are many secreted vesicles. The cells include one or two dark spots. In the internal yellow body, most cells are distributed at moderate density near the bottom of the colony, and others are sparsely embedded in EPM in the intermediate layer as shown earlier [38]. In contrast, the top colony surface is almost cell-free and covered by fibers and vesicles. Dark spots of high electron density are rarely observed or only faintly visible in the cells. (Right panel) *sprB* nonspreading colony. In contrast to the WT, the *sprB* cells are densely packed in the nonspreading yellow colonies. The somewhat homogenous top surface is almost cell-free and covered by fibers and vesicles, which is similar to the surface of the WT colony body.

The data presented extend the study reported in Sato et al. [38]. The combined results lead to the schema for *F. johnsoniae* biofilm expansion by gliding motility shown in Figure 11. In the first part of this study (Figure 1, Figure 2 and Figure 3 of [38]), epon-embedding and thin-sectioning revealed that the cell density and EPM in the translucent fringe of spreading WT colonies is completely different from the cell density and EPM towards the bottom of the yellow colony body. Further, time-lapse fluorescence microscopy visualized small cell clusters followed by cells at the leading edge (Figure S2a of [38]). However, it was not clear what led to the outermost cell clusters. In the present study, grid-stamp negative-stain TEM showed that single leading cells lay a path of EPM outside the translucent leading edge of the colonies (Figure 1c and Figure 11). At the translucent edge, cells make head-to-tail and/or side-to-side contact and are surrounded by thin and thick fibers to form a cluster (Figure 3). The cells are also attached to and surrounded by many vesicles, which is in good agreement with the first part of this study suggesting that the cells near the leading edge secrete many vesicles [38]. In more proximal (inside) surface regions of the colony, more cells made head-to-tail and/or side-to-side contact forming clusters that were larger than those at the periphery (Figure 1 and Figure 4a–c). The cells were in close contact, and one of their ends (poles) was sometimes deformed to fit to the neighboring cells (Figure 4a–e). In addition, they were further surrounded by poorly stained filamentous matrices. These results suggest that all the cells in a cluster move in the same direction [38]. The top of the thick colony body was almost cell-free, but it was covered by EPM, including a filamentous network interspersed with thick fibers and vesicles (Figure 1f, Figure 4d–f and Figure 11); in agreement, the first part of this study showed that most cells are embedded in EPM deep inside the colony body, close to the agar layer [38].

Colony morphology might largely depend on EPM formation in many biofilm-forming bacteria. Single *F. johnsoniae* cells laid a path of filaments outside the colony edge (Figure 1c), and the neighboring cells at the colony edge made head-to-tail and/or side-to-side contact forming cell clusters with filamentous intercellular connections (Figure 1, Figure 2 and Figure 3). Because the specific paths left by such clusters were followed by other cell clusters (Figure 1c, upper and Figure S2a of [38]), the guides (sign-posts) for cell gliding and further colony spreading might be EPM, which thus also regulates the colony morphology.

EPM secreted on the culture substrate (Figure 1, Figure 2, Figure 3 and Figure 4) might both determine the direction that gliding cells move and induce biofilm formation. Adhesion between the bacterium and proteins on the medium surface is suggested to be important for the gliding mobility and biofilm formation on 1% A-PY2 [3]. It would be interesting to see whether the bacterial cells are attached directly to the 1% A-PY2 or to a self-secreted substance including proteins and glycans attached to the agar. Because the biofilm-related social motility of the model system *F. johnsoniae* seems to be relevant to the motility of other microorganisms with similar characteristics, biofilms formed by different bacterial species with gliding motility should be further studied using the grid stamp method to understand their structures.

Biofilm-induced antibiotic resistance [34] might be related to the structures revealed here. The antibiotic resistance of various biofilm-forming bacteria is known to increase when they form biofilms [34]. The following three mechanisms have been proposed to explain this: (1) limited penetration of the antibiotic; (2) altered chemical microenvironment within the biofilm; (3) subpopulations of micro-organisms in a biofilm [34]. Because the biofilm-forming ability (Figure 8) was related to the antibiotic resistance (Figure 10) of *F. johnsoniae*, poor penetration of antibiotics across the biofilm matrix might be realized by the clogged filter-like structure comprised of abundant fibers and vesicles at the surface (Figure 4b) and inside [38] of colonies, which could greatly reduce the number of cells targetable by antibiotics. It is similar to the dense EPM with a thin backbone network formed by *Staphylococcus* species [18,34]. Further precise analysis of drug permeability through the biofilm using immuno-EM [18,19] is awaited. In our study, grid stamp TEM and uranyl acetate staining revealed that bacteria with one or two dark spots have a specific spatial distribution near the translucent edge of the biofilm colony (Figure 2a,c and Figure 3) but not elsewhere, suggesting that the biofilm includes multiple cell subpopulations. Such differences between cells might contribute to drug resistance. Both of *F. johnsoniae* WT and *remA* mutant form spreading colonies on PY2 medium [7]. Since these strains produced spreading colonies in the inhibition zone of the tetracycline disc diffusion test on PY2 medium (Figure 10), they left from the viable but non-culturable state earlier than the other strains to form the colonies in the presence of antibiotics. These suggest that the ability to spread colonies, rather than the capability to secrete proteins, contributes to antibiotic resistance of the colony. These results also suggest that gliding motility has an advantage for biofilm expansion even in the presence of antibiotics.

Although we could not identify what the dark spots in WT cells were, such dark spots were not observed in the colonies formed by *sprB* (Figure 2b,d and Figure 5) and *gldK* (Figure 6b–e) mutant *F. johnsoniae* cells. SprB is delivered to the cell surface by the T9SS. This suggests that their formation requires at least SprB-dependent gliding motility. Such dark spots were hardly observed in the colonies formed by *gldG* mutant cells (Figure 7b,c). In *F. johnsoniae*, disruption of the *gldG* gene resulted in normal levels of *gldJ* transcript but decreased levels of GldJ protein, which is required for gliding motility [14]. These suggest a working hypothesis that the formation of the dark spots requires the gliding motility-associated protein GldJ. Because areas highly dense with gliding motility-related complexes were observed as focal adhesin complexes in *Myxococcus xanthus* cells [41], the dark spots we observed might be focal adhesion complexes that include the adhesin SprB. Further study is awaited to understand whether they are related to cell activities, including cell motility.

The methodology presented here could also be applied to study biofilm-related chronic diseases. Many chronic diseases are attributable to biofilm formations, which are sometimes associated with cystic fibrosis or surgical implants and catheters [42,43,44,45]. Multiple diseases, including systemic diseases, can be caused by biofilm-associated pathogens (e.g., in endocarditis by pathogens such as *Streptococcus* sp. and *Staphylococcus aureus*; in periodontitis by pathogens such as *P. gingivalis.*) [44,45]. *P. gingivalis* is considered to be the key periodontal pathogen involved in the development of periodontitis [46]. Infectious oral diseases, such as dental caries and periodontitis, are sometimes caused by biofilms called dental plaques [47,48]. Each plaque in the oral cavity is estimated to contain more than 500 bacterial species, including gliding bacterium species [49]. The grid stamp method can be applied not only to the biofilms formed at the interface between air and wet substrates but also to biofilms formed in liquid, e.g., deep in periodontal pockets, and to soft materials. Because of its speed, the method would assist clinical diagnosis. Developments, including the use of a robust SiN-film grid [50,51] would make it possible to obtain a stamp directly from oral cavity areas thought to be affected by biofilm or dental plaques.

The Grid Stamp-Peel method could also be adapted to allow liquid-phase EM, e.g., atmospheric scanning electron microscopy (ASEM), making it possible to image biofilms, dental plaques, or other soft specimens peeled off by the stamp in liquid, while avoiding the drying step required for negative-stain TEM. For example, the TEM grid used for stamping could be replaced by a SiN-film windowed ASEM dish and the surface structure immersed in aqueous liquid imaged using the inverted SEM employed in this method. The same technique could be applied to various wet organic and inorganic samples [35,39,50,51].

## 4. Materials and Methods

### 4.1. Bacterial Strain and Biofilm Cultivation

*F. johnsoniae* strains were grown in casitone-yeast extract (CYE) medium at 24 °C (Becton, Dickinson and Co., New Jersey, USA). The details of the bacterial strains and plasmids used are shown in Table 1 [7,52,53,54].

To observe colony spreading, *F. johnsoniae* WT, *sprB* deletion mutant CJ1922 (*sprB*) cells, spontaneous *gldK* mutant UW102-57 (*gldK*), and spontaneous *gldG* mutant UW102-34 (*gldG*) were grown in CYE medium at 27 °C with shaking (175 rpm) overnight. The cells were collected as a pellet by centrifugation at 800× *g* for 10 min at 22 °C. The pellet was resuspended in the same volume of washing buffer (10 mM Tris-HCl pH 7.4) by vortexing, and the suspension was centrifuged at 800× *g* for 10 min at 22 °C. These steps were repeated twice. The cells were spotted onto peptone yeast (PY2) agar medium (peptone and yeast extract: Becton, Dickinson and Co., agar: Ina Food Industry Co., Ltd., Nagano, Japan) in a dish 9 cm in diameter and incubated at 24 °C for 5 days [39].

### 4.2. Carbon-Grid Stamp-Peel Method

Thin flat carbon film supported by a copper mesh grid was rendered hydrophilic by glow discharge in a reduced atmosphere of air (ca 1 Pa) at 6 mA for 90 sec using a PIB-20 ion sputter (Vacuum Device Inc., Ibaraki, Japan). Colony surface was lightly pressed (stamped) by the glow-discharged carbon film grid, and some surface cells and matrix structures of the colony were removed on the film grid. Sample on the film was washed with six drops of double distilled water (DDW). The sample side of the grid was placed onto a drop of 2.0% uranyl acetate for 30 s twice and dried in air.

### 4.3. TEM Imaging

The negatively stained sample grids as described above were observed and recorded with a JEM1230 TEM (JEOL, Tokyo, Japan) at an acceleration voltage of 100 kV, equipped with Orius SC200 or Bioscan CCD cameras (GATAN, Pleasanton, CA, USA).

### 4.4. Crystal Violet Biofilm Assay

Three hundred microliters of 100-fold diluted overnight culture was added per well in 24-well assay plates and incubated for 24 h at 25 °C. After removing planktonic bacteria from the plate, biofilms were evaluated by the crystal violet assay. An amount of 0.3 mL of 0.5% crystal violet was added to each well of the plate. The plate was incubated for 30 min at 25 °C before removing the staining solution, and then it was washed three times with 350 μL phosphate-buffered saline (PBS, pH 7.5). After removing the washing solution, 300 μL 96% EtOH was added per well to dissolve the biofilm-bound crystal violet by gently knocking the plate. Absorbance was measured at 595 nm.

### 4.5. Disk Diffusion Susceptibility Test

*F. johnsoniae* WT and the mutants were subjected to antimicrobial susceptibility testing using the Kirby–Bauer disk diffusion method. Strains were grown in CYE medium at 27 °C with shaking (175 rpm) overnight. The cells were centrifuged at 800× *g* at 22 °C for 10 min and collected as a pellet. The pellet was resuspended in the same volume of washing buffer (10 mM Tris-HCl pH 7.4) by vortexing, and the suspension was centrifuged at 800× *g* at 22 °C for 10 min. These steps were repeated. The suspension was adjusted to 0.2 at OD_600_ and inoculated onto PY2 and CYE agars. The tetracycline disc (30 μg, BD-Sensi Disc^TM^) was placed on the surface of the prepared medium, and the plates were incubated at 25 °C for 48 h. Thereafter, the inhibition zone diameters were measured.

## Figures and Tables

**Table 1:** Strains used in this study.

Strain	Description	Reference
Cj1827	Wild-type *rpsL2*	[52]
UW102-57	Spontaneous *gldK* mutant	[53]
CJ1300	*gldL*::*HimarEm1*	[54]
FJ113	Spontaneous *gldM* mutant	[54]
CJ1631A	*gldNO*::*HimarEm1*	[54]
UW102-55	Spontaneous *gldJ* mutant	[53]
CJ1922	*sprB* deletion mutant	[52]
FJ149	Spontaneous *sprE* mutant	[54]
UW102-34	Spontaneous *gldG* mutant	[53]
UW102-41	Spontaneous *gldI* mutant	[53]
CJ1984	*remA* deletion mutant	[7]

## Abbreviations

WT	Wild-type
*sprB*	*sprB* deletion mutant CJ1922
*gldK*	Spontaneous *gldK* mutant
*gldL*	*gldL*::*HimarEm1*
*gldM*	Spontaneous *gldM* mutant
*gldNO*	*gldNO*::*HimarEm1*
*gldJ*	Spontaneous *gldJ* mutant
*gldG*	Spontaneous *gldG* mutant
*gldI*	Spontaneous *gldI* mutant
*sprE*	Spontaneous *sprE* mutant
*remA*	*remA* deletion mutant
T9SS	Type IX secretion system
EPM	Extracellular polymeric matrix
TEM	Transmission electron microscopy
